# Decreased Incidence of Acute Pancreatitis in Finland with Biliary Etiology Predominant in Initial Episodes: A Population-Based Study

**DOI:** 10.1007/s10620-025-09625-4

**Published:** 2025-12-29

**Authors:** Anssi Nikkola, Jussi Nikkola, Elisa Kari, Aki Roponen, Anna Tapaninaho, Juhani Sand, Johanna Laukkarinen

**Affiliations:** 1 Department of Gastroenterology and Alimentary Tract Surgery, Tampere University Hospital, Tampere, Finland; 2 Faculty of Medicine and Health Technology, Tampere University, Tampere, Finland

**Keywords:** Acute alcoholic pancreatitis, Acute pancreatitis, Etiology, Finland, Incidence

## Abstract

**Background and Aims:**

In the 1990s, the incidence of acute pancreatitis (AP) in Finland exceeded 70 per 100,000 inhabitants, with alcohol as the etiology in 70% of cases, whereas in most other countries biliary etiology predominates. Diagnostic methods have since improved, and more recent studies have already reported lower incidence rates and a different etiologic distribution in Finland. We report a population-based study on the current incidence and etiology of AP in Pirkanmaa Finland.

**Methods:**

All patients treated for AP at Tampere University Hospital during 2014–2015 were identified from hospital records. Patient demographics and etiology-related data were collected.

**Results:**

The Pirkanmaa Hospital District population during the study period averaged 525,926. A total of 558 patients were treated for AP, comprising 622 episodes and yielding an incidence of 59.1 per 100,000 inhabitants. Of these, 451 patients (72.5%) presented with a first episode, corresponding to an incidence of 42.9 per 100,000. Among first episodes, etiology was biliary in 36.8% and alcoholic in 29.4%. For all episodes, biliary and alcoholic etiologies accounted for 32.5% and 34.1%, respectively. Other causes included post-ERCP (2.7%), tumor (3.2%), drug-induced (2.4%), autoimmune (1.4%), post-operative (1.0%), hypertriglyceridemia (0.6%), hypercalcemia (0.2%), SPINK1-associated (0.8%), miscellaneous (0.3%), idiopathic (10.6%), and not reliably determined (10.1%).

**Conclusion:**

The total incidence of AP in Pirkanmaa, Finland, was 59.1 per 100,000, and first-episode AP 42.9 per 100,000 – lower than historically reported and comparable to other Western countries. Biliary etiology was most common in first episodes, indicating that alcohol is no longer the predominant etiology of AP in the study area in Finland.

## Introduction

The incidence of acute pancreatitis (AP) in most European countries as well as in the United States is around 30–50/100,000 inhabitants [1, 2]. The pooled global incidence of AP has been reported to be 34/100,000 In Western countries the most common etiology is gallstones accounting for approximately 30–70% of cases, with alcohol in second place with 20–40% of cases [3–5]. Some countries, however, do have a higher percentage of alcohol-related AP. For example, in India approximately 45% of cases are attributed to acute alcoholic pancreatitis (AAP) [5]. In Finland, the incidence of AP has been reported to be between 46.6 (1970) and 73.4 (1989)/100,000 inhabitants and alcohol has been considered the predominant etiology with 70% of cases [6]. However, the evidence is based on a 1993 register-based study by Jaakkola et al., which examined the incidence of pancreatitis in Finland during 1970–1989. In the following decades, considerable progress has been made in imaging modalities, laboratory testing, as well as in classifications and guidelines in AP. For example, computed tomography (CT) as an imaging modality was widely introduced for clinical use in the 1980s. At that time, the quality, availability, and feasibility of CT scan as a diagnostic tool for AP were far from the level of present standards. The first Atlanta Criteria for the classification of acute pancreatitis were introduced in 1993 and revised in 2012 [7, 8]. Hypercalcemia, hypertriglyceridemia, autoimmune pancreatitis, and drug-induced pancreatitis were not well defined or routinely studied etiologies for AP in 1970–1989. Recently, Belfrage et al. (2023) reported a hospital-based study from Helsinki with an AP incidence of 42.2 per 100,000, in which alcohol accounted for 46.8% of cases and gallstones for 23.4% [9].

Alcohol consumption has changed in recent decades in Finland. According to the Finnish Institute for Health and Welfare (THL) registry, in 1970–1989 annual alcohol consumption was estimated at around 7–10 L per person (100% alcohol) [10]. In the 2010s, annual alcohol consumption was reportedly around 9–11 L per person, with a declining trend descending from peak annual alcohol consumption of around 12 L per person in the late 2000s. In 2020 annual alcohol consumption was estimated at 9.2 L per person.

Hence, there is clearly a need to update the incidence and distribution of etiologies in AP in Finland. Our aim was to investigate this in a population-based study in Pirkanmaa hospital district (nowadays called the Wellbeing Services County of Pirkanmaa), which was the second largest hospital area in Finland.

## Methods

This study was population-based within the Pirkanmaa Hospital District, as Tampere University Hospital and its affiliated units were the sole providers of AP care in this region during the study period (January 1, 2014, to December 31, 2015). Thus, all AP cases in the catchment population were captured. ICD-10 codes studied were K85 (AP) as well as K86 (other diseases of the pancreas) to minimize the risk of under-reporting. From the hospital records we collected data on AP etiology, particularly the clinical diagnoses made by clinicians, as well as patient histories, results from imaging studies and etiologic laboratory testing. Baseline characteristics and information on possible recurrences were also collected. The diagnosis of AP required at least two of the following three findings: 1) typical epigastric pain 2) elevated plasma amylase at least three times above the normal upper limit and 3) typical imaging findings of AP (ultrasonography/CT/magnetic resonance imaging (MRI)) according to the revised Atlanta Classification [10]. Patients clinically diagnosed with chronic pancreatitis were excluded from the study.

Etiology of AP was determined using information on patient history regarding possible previous AP episodes and their etiology, alcohol consumption, history of gallstones, use of drugs and medications, recent invasive procedures (especially ERCP), history of autoimmune disorders as well as family history of AP, laboratory serum tests (i.e., amylase and liver enzymes, calcium, triglycerides), and imaging studies [11]. Etiology was considered alcoholic when patients had a documented history of sustained heavy alcohol consumption or binge drinking consistent with international consensus definitions supported by clinical evaluation and, when available, AUDIT scores. Diagnosis required sufficient exclusion of other potential etiologies through imaging and laboratory testing. Thus, AAP was not based on any alcohol use alone, but on a consistent harmful pattern of use in combination with exclusion of alternative causes [12]. Biliary AP (BAP) was determined as the etiology by sufficient elevation in liver enzymes according to IAP/APA 2013 guidelines (elevation > 150 U/L in alanine aminotransferase within 48 h after onset of symptoms as well as elevated alkaline phosphatase and/or bilirubin are also suggestive of biliary etiology) and presence of gallstones/dilated bile ducts in imaging studies (transabdominal ultrasound, computed tomography and/or magnetic resonance cholangiopancreatography) after exclusion of other potential etiologies [11]. Other, more rare etiologies were determined clinically. When no etiology was found despite comprehensive evaluation (during hospital stay or at a follow-up visit) of risk factors and causes, etiology was deemed idiopathic. Diagnostics of idiopathic pancreatitis included exclusion of high calcium and triglyceride levels, sufficient imaging studies (preferably magnetic resonance cholangiopancreatography), consideration of possible medication-related AP and determination of immunoglobulins and genetic testing in selected cases [13]. When no etiology was determined during hospital stay or during out-patient follow-up visit after hospitalization and sufficient etiologic studies were not conducted, etiology was deemed to be not reliably determined.

To calculate the incidence of AP and its various etiologies, we estimated the average population size in 2014–2015 in Pirkanmaa Hospital District using data from the Statistics Finland [14]. During the study years, Tampere University Hospital system, consisting of main hospital and its subsidiary hospitals (Tampere University Hospital Valkeakoski and Tampere University Hospital Hatanpää) was the only facility treating AP patients in the catchment area.

Statistical analysis was carried out using IBM SPSS Statistics for Macintosh and Windows (Version 28.0, Armonk, NY). P-values under 0.05 were considered statistically significant. Chi-square and Fisher’s exact test as appropriate were used to test for statistical significance.

This study was performed according to the Helsinki Declaration and was approved by the institutional review board of the Pirkanmaa Hospital District (R16035).

## Results

According to Statistics Finland, the average population in Pirkanmaa Hospital District was 525,926 inhabitants during the years 2014–2015 [13]. From this population, 558 patients were treated for AP, experiencing a total of 622 AP episodes during the study period. The yearly incidence of AP was 59.1 per 100,000 inhabitants. Of the patients 451 (72.5%) suffered their first AP. The annual incidence of the first AP was 42.9 per 100,000 inhabitants. Of these patients 37.3% were female, and mean age was 60 years, as shown in Table 1. Etiology was reliably determined during hospitalization in 89.9% of cases.

**Table 1:** Baseline characteristics of 558 patients with acute pancreatitis

Variable	Patients total n = 558
Sex, male (%)/female (%)	350 (62.7)/208 (37.3)
Mean age (range), years	59.8 (7–97)
Smoking (%)	
Yes	156 (28.0)
No	236 (42.3)
Not recorded (NR)	166 (29.7) (30.5)
Smoking in AAP Yes/No/NR (%)	73/24/85 (40.1/13.2/46.7)
Smoking in BAP Yes/No/NR (%)	21/108/50 (11.7/60.3/27.9)

*AAP* = acute alcohol pancreatitis, *BAP* = biliary acute pancreatitis

**Fig. 1:**
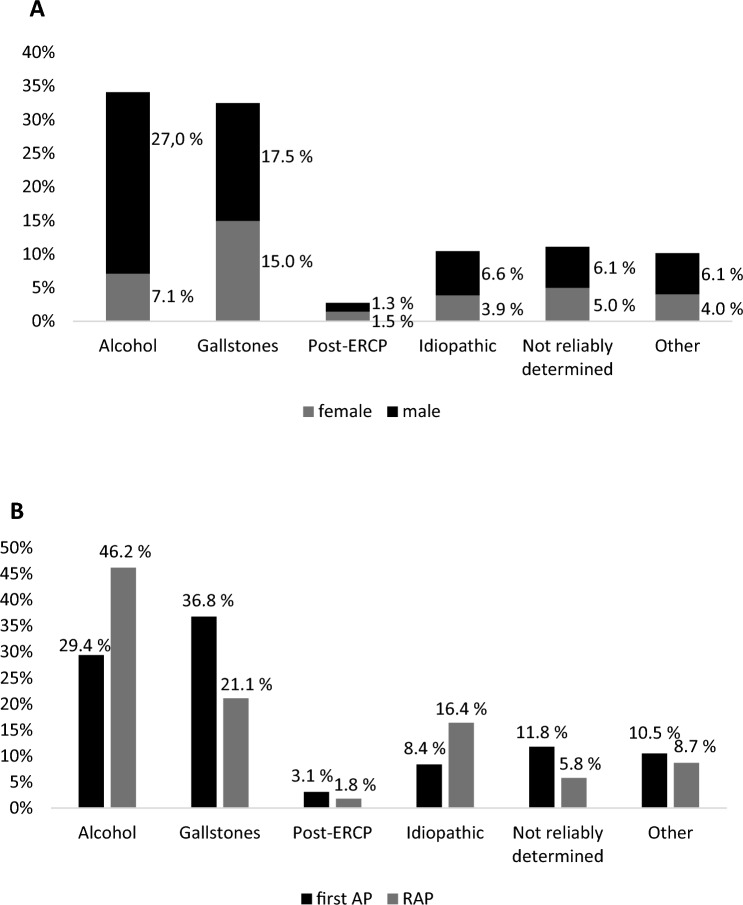
Distribution of different etiologies **A** in overall acute pancreatitis (AP) episodes stratified by sex (n = 622). **B** in first AP (n = 451) and recurrent AP (RAP) (n = 171)

Out of all episodes (including recurrent episodes), the overall etiology was biliary in 202 (32.5%) and alcoholic in 212 (34.1%) of cases (Fig. 1 and Table 2). In women, etiology of AP was biliary in 41.1% and alcoholic in 19.5% of the cases with a statistically significant difference compared to men (BAP 27.5% and AAP 42.4% respectively, p < 0.001 in both) (Table 2). Other etiologies included post-ERCP in 2.7%, tumor in 3.2%, drug-induced in 2.4%, autoimmune in 1.4%, post-operative in 1.0%, hypertriglyceridemia in 0.6%, hypercalcemia in 0.2%, SPINK1 associated in 0.8%, miscellaneous in 0.3%, and idiopathic in 10.6% (Table 2). In 63 patients (10.1%) etiology was not reliably determined or studied during hospitalization or on possible follow-up visits (Table 2).

**Table 2:** Etiologies of 622 acute pancreatitis (AP) episodes in female and male patients in first and recurrent pancreatitis

		Etiologies in female and male (all AP patients)			Etiologies in first and recurrent AP			Etiologies in female and male (first AP patients)		
AP etiology, n = 622	Number of episodes (%)	Female; n = 226 (%)	Male; n = 396 (%)	p-value	First AP; n = 451 (%)	RAP; n = 171 (%)	p-value	Female; n = 172 (%)	Male; n = 280 (%)	p-value
Alcohol	212 (34.0)	44 (19.5)	168 (42.4)	< 0.001	133 (29.4)	79 (46.2)	< 0.001	30 (17.4)	103 (36.8)	< 0.001
Gallstones	202 (32.5)	93 (41.1)	109 (27.5)	< 0.001	166 (36.8)	36 (21.1)	< 0.001	76 (44.2)	90 (32.1)	0.082
Post-ERCP	17 (2.7)	9 (4.0)	8 (2.0)	0.156	14 (3.1)	3 (1.8)	0.364	7 (4.1)	7 (2.5)	0.365
Tumor	20 (3.2)	9 (4.0)	11 (2.8)	0.258	15 (3.3)	4 (2.3)	0.387	6 (3.5)	10 (3.6)	0.964
Drug-induced	15 (2.4)	6 (2.7)	9 (2.3)	0.781	14 (3.1)	1 (0.6)	0.069	6 (3.5)	8 (2.9)	0.716
Hypertriglyseridemia	4 (0.6)	0 (0)	4 (1.0)	NA	4 (0.9)	0	NA	0 (0)	4 (1.4)	NA
Hypercalcemia	1 (0.2)	0 (0)	1 (0.3)	NA	1 (0.2)	0	NA	0 (0)	1 (0.4)	NA
Post-operative	6 (1.0)	2 (0.9)	4 (1.0)	0.868	6 (1.3)	0	NA	2 (1.2)	4 (1.4)	0.813
Autoimmune	9 (1.4)	4 (1.8)	5 (1.3)	0.622	5 (1.1)	4 (2.3)	0.245	2 (1.2)	3 (1.1)	0.929
SPINK1 mutation	5 (0.8)	4 (1.8)	1 (0.3)	0.043	1 (0.2)	4 (2.3)	0.008	1 (0.6)	0 (0)	NA
Sfincter of Oddi dysfunction	1 (0.2)	0 (0)	1 (0.3)	NA	0	1 (0.6)	NA	0 (0)	1 (0.3)	NA
Groove pancreatitis	1 (0.2)	1 (0.4)	0 (0)	NA	1 (0.2)	0	NA	0 (0)	0 (0)	NA
Idiopathic	66 (10.6)	25 (11.1)	41 (10.4)	0.817	38 (8.4)	28 (16.4)	0.004	15 (8.7)	23 (8.2)	0.863
Not reliably determined	63 (10.1)	31 (13.7)	32 (8.1)	0.028	53 (11.8)	10 (5.8)	0.032	27 (15.7)	26 (9.3)	0.069

*RAP* = recurrent acute pancreatitis

In first-episode AP, the most common etiology was biliary (36.8%), followed by alcohol (29.4%), with the difference being even more pronounced among female patients (44.2% vs. 17.4%, respectively). In men, however, alcohol remained the leading etiology in first episodes (36.8% vs. 32.1%, respectively) (Table 2). In recurrent acute pancreatitis (RAP), alcohol was the most common etiology with 46.2% of cases and biliary with 21.1%, with a statistically significant difference compared to first AP (Table 2). Furthermore, idiopathic and SPINK1-associated AP was statistically significantly more prevalent with RAP as an etiology compared to first AP. Etiology was not reliably determined significantly more often in first AP than in RAP (Table 2).

Etiology of AP was significantly more often alcohol in men than in women (42.4% vs. 19.5%, p < 0.001). Conversely, BAP was significantly more common in women than in men (41.1% vs. 27.5%, p < 0.001). Women also had more SPINK1 mutation-related AP (1.8% vs. 0.3%, p = 0.043) and not reliably determined etiology for AP compared to men (13.7% vs. 8.1%, p = 0.028) (Table 2).

In AAP patients, the mean AUDIT score was 17 (range 3–37, n = 25); these patients consumed on average 9.5 daily portions of alcohol prior to the AP episode (range 2–40, n = 25). Of the AP patients 39.7% (392) were smokers (Table 1). The difference in smoking between AAP and BAP patients was significant (75.3% vs. 16.3%, p < 0.001). In patients with hypertriglyceridemic AP, mean triglyceride level (mmol/L) was 36.5 (range 12–78, n = 4) and in a single patient suffering from hypercalcemia associated AP, ionized calcium level (mmol/L) was 2.07. In the patients with drug induced AP (n = 15) the drugs suspected were mercaptopurine (3 patients), PEG-asparaginase (2), simvastatin (2), atorvastatin (1), azathioprine (1), terbinafine (1), methotrexate (1), sitagliptin (1), imatinib (1), clozapine (1), gemsitabine/nab-paclitaxel (1). During the years 2014–2015 a total of five children (< 18 years of age) were treated for AP. Their mean age was 13 (range 5–16). Etiologies were one biliary, two drug-induced (associated with PEG-asparaginase, both acute lymphocytic leukemia patients), one idiopathic and one not reliably determined.

In-hospital mortality in AAP during the years 2014–2015 in the study area was 2.9% (18 out of 622 AP episodes). The etiology of AP in these cases were 6 alcohol, 8 biliary, and 4 not reliably determined. All-cause 90-day mortality was 4.7% (29 out of 622 AP episodes).

## Discussion

The incidence of AP in Finland was considered for decades to be over 70/100,000 inhabitants, with alcohol the predominant etiology at around 70% of cases. In recent decades, diagnostic capabilities have improved dramatically. In this study we updated the population-based incidence and etiologic distribution in AP in Finland and the study area to the twenty-first century. We report a total AP incidence of 59.1 per 100,000 inhabitants and first AP incidence of 42.9 per 100,000 inhabitants. AAP and BAP each account for about one-third of cases, similarly to reports from other European countries (Roberts et al. Pancreatology 2017). In first AP, BAP accounts for 36.8% and AAP for 29.4% of cases.

Compared with the study by Jaakkola et al., who reported an incidence of AAP of 51.4 per 100,000 inhabitants in 1989, we observed an incidence approximately 61% lower in our study [6]. Interestingly, alcohol consumption in our study period was higher than in the period 1970–1989 (9–11 vs. 7–10 L per person, 100% alcohol) [10]. Binge drinking has been speculated to play a role in the more frequent development of AP [15]. Drinking habits in Finland have been speculated to be changing as alcohol consumption has been decreasing since 2007 [16]. Furthermore, in Finland young adults drink more mild beverages and the binge drinking pattern has likewise been shown to be slightly decreasing. However, we attribute the decrease in AAP cases largely to the better diagnostic evaluation and pathways in establishing the etiology of AP. At the time of the study by Jaakkola et al. (study years 1970–1989), the absence of comprehensive etiologic evaluation likely led to many cases being misclassified as alcoholic acute pancreatitis (AAP) in Finland. In modern practice, however, the diagnostic approach to AP is more systematic and includes a detailed history (e.g., alcohol consumption, past gallstones, hypertriglyceridemia or hypercalcemia, medications, prior surgeries or procedures, concomitant autoimmune diseases, and patient/family history of pancreatitis), laboratory testing (triglycerides, calcium levels, liver enzymes), and imaging (ultrasonography, computed tomography, and/or magnetic resonance imaging as appropriate). When no clear etiology is identified despite this work-up, additional investigations such as autoimmune markers or genetic testing may be pursued [11]. In parallel, demographic changes must also be considered: in Finland, as in many Western countries, the population is gradually aging, with the proportion of individuals aged ≥ 65 increasing from 16 to 22% between 2005 and 2018 [17]. Since gallstone prevalence rises substantially with age, whereas AAP is more frequent among younger individuals, this demographic shift may partly explain the observed change in etiology from alcohol toward biliary disease [18]. Our study demonstrates clear sex-related differences in the etiology of AP: alcohol was significantly more common in men, whereas gallstone-related AP predominated in women. This pattern is consistent with prior European reports, where men more often engage in heavy episodic drinking, while gallstone disease is more frequent in women [4, 19].

The strengths of our study include the accuracy of our data, as each case in the hospital records was individually verified and scrutinized in contrast to register-based studies. The Finnish Institute for Health and Welfare (THL) registry regarding population sizes in each area is known to be quite comprehensive and is thus unlikely to lead to biased evaluation of the incidence in the study area. At the time of the study Pirkanmaa Hospital District was the second largest in Finland covering almost 10% of the total population. We estimate that the results presented in this study may provide a reasonable approximation of the national incidence and etiologic distribution of AP in Finland. A hospital-based study from Helsinki, Finland recently investigated the incidence of acute pancreatitis (2016–2018) [9] These researchers reported a total incidence of AP of 42.2/100,000 inhabitants. Alcohol was reported as the main etiology with 46.8% of cases. Gallstones accounted for 23.4%, post-ERCP 2.9%, idiopathic 20.7% and other 6.2%. Belfrage et al. reported comparable results regarding the incidence of AP, but this study was not population-based. As previously mentioned, the Tampere University Hospital network was the only hospital treating AP in its catchment area during the study years. Furthermore, we determined the etiology as idiopathic when comprehensive etiologic studies were conducted, in many cases on a subsequent follow-up visit. If the etiologic studies were not complete or sufficiently evaluated by a clinician, we reported etiology to be not reliably determined.

The main weakness of our study is that in a proportion of AP cases the etiology could not be reliably determined, either during or after hospitalization, nor from patient files. These patients were categorized as ‘not reliably determined.’ Most had mild symptoms and were discharged after brief observation, and in many cases, AP was a secondary rather than primary diagnosis. Nevertheless, ten RAP cases were observed in this group. In AP, and especially in RAP, accurate etiologic classification is essential for preventing recurrence, and this has been increasingly emphasized in our facility. Idiopathic AP accounted for about 10% of patients and was significantly more frequent in RAP compared with first AP (16.4% vs. 8.4%), which was expected and is consistent with incidences reported in other Western countries [13]. Another limitation is that very mild cases not presenting to emergency care may have been missed. However, in Finland patients with suspected AP are routinely referred to hospital emergency departments, and during the study period Tampere University Hospital was the sole provider of such care in the region. Thus, we are confident that virtually all clinically significant cases were captured.

The prevalence of smoking in Finland was reportedly 17% in 2015 (age group 20–64 years), with a clearly declining trend over the years [20]. In our study, smoking was reported in 39.8% of AP patients when recorded. In AAP smoking was even more common among patients and was significantly more prevalent than in BAP patients. Smoking is a known risk factor for AP and especially in AAP [21]. Smoking has also been linked to greater amounts of alcohol consumed. AAP has been shown to be significantly more prevalent in men and BAP in women, which was also shown in our study [22, 23]. No obvious explanation can be found for the gender differences in SPINK1 related AP nor for the fact that AP etiology was not reliably determined more often in women than in men. However, only five patients in our study had SPINK1 mutation associated AP.

Including the data presented here, the incidence of first AP is reported at around 42.9/100,000 in Finland, 38/100,000 in Sweden, 23/100,000 in Norway and 38/100,000 in Denmark, with alcohol-induced pancreatitis in around 29.4%/17%/17%/30% and gallstones as the etiology in around 36.8%/

49%/49%/44% of the cases [24–26]. Average alcohol consumption has been reported at 7.2 L/year in Finland, 7.2 L in Sweden and 6.1 L in Norway during the study years 2014–2015 (100% alcohol consumption in > 15-year-olds) [27]. However, according to the Finnish Institute for Health and Welfare registry annual alcohol consumption in Finland in the 2010s was 9–11 L per person when accounting for personal importation from abroad, which is popular in Finland [9]. Furthermore, Finland still has a high rate of heavy episodic drinking, and these observations may explain the higher incidence of AAP in Finland compared to Sweden and Norway [16]. AAP has a high tendency for recurrences (34–46%) when abstinence is not achieved after the initial episode [28, 29]. This finding explains the higher proportion of AAP in total incidence of AP versus in the first episode shown in our study. Repeated interventions against alcoholism after an episode of AAP have been shown to reduce recurrences effectively [30].

## Conclusion

The population-based incidence of AP in Pirkanmaa, Finland, is 59.1/100,000 overall and 42.9/100,000 for the first episodes, both lower than historically reported. Among the first AP episodes, biliary etiology is the most common, and among all episodes biliary and alcohol etiologies are equally common. Alcohol is therefore no longer the predominant etiology in the study area in Finland.

## Data Availability

The datasets generated and analyzed during the current study are available from the corresponding author on reasonable request.
